# Real‐world benefit of combination palbociclib and endocrine therapy for metastatic breast cancer and correlation with neutropenia

**DOI:** 10.1002/cam4.4295

**Published:** 2021-09-30

**Authors:** James Sun, Xiaojun Zhong, Junjie Ma, Weihong Sun, Hyo S. Han, Hatem H. Soliman, Loretta S. Loftus, Ricardo L. B. Costa, Avan J. Armaghani, Aixa E. Soyano‐Muller, Brian J. Czerniecki, M. Catherine Lee, John V. Kiluk, Nazanin Khakpour, Susan J. Hoover, Christine Laronga, Hung T. Khong

**Affiliations:** ^1^ Department of Breast Oncology Moffitt Cancer Center Tampa Florida USA; ^2^ Department of Pharmacotherapy University of Utah Salt Lake City Utah USA; ^3^ Present address: Depart of Surgery University Hospitals Cleveland Medical Center Cleveland Ohio USA; ^4^ Present address: Department of Medical Oncology The First Affiliated Hospital of Nanchang University Jiangxi China; ^5^ Present address: Amgen Inc. Thousand Oaks CA USA

**Keywords:** absolute neutrophil count, endocrine therapy, metastatic breast cancer, neutropenia, neutrophil–lymphocyte ratio, palbociclib

## Abstract

**Background:**

Combination CDK4/6 inhibitor and endocrine therapy has been shown to significantly improve progression‐free survival (PFS) in patients with hormone receptor (HR)‐positive, HER2‐negative metastatic breast cancer (mBC). The aim of this retrospective study was to evaluate the real‐world benefit of first‐line combination therapy in this cohort and to correlate treatment efficacy with neutropenia, a common toxicity of CDK4/6 inhibitors.

**Methods:**

This study included HR‐positive, HER2‐negative advanced or mBC patients who were treated with palbociclib plus endocrine therapy, mainly letrozole, between 1 January 2015 and 1 March 2018. Progression‐free survival (PFS) was determined using Kaplan–Meier analysis. The predictive value of absolute neutrophil count (ANC) and neutrophil‐to‐lymphocyte ratio (NLR) for PFS were explored using Cox regression models. Both ANC and NLR were used as a time‐dependent variable.

**Results:**

In total, 165 patients were included with median PFS of 24.19 months (95% CI 18.93–NR). Median PFS for patients with bone‐only metastases (*n* = 54) was not reached (95% CI 18.21–NR). Among patients with all other metastases (*n* = 111), median PFS was 24.19 months (95% CI 16.33–33.82). Lower ANC was correlated with decreased risk of progression (HR 0.84, 95% CI 0.71–0.97, *p* = 0.008). There was no significant association between NLR and the risk of disease progression (HR 1.07, 95% CI 0.97–1.18, *p* = 0.203).

**Conclusion:**

The effectiveness of palbociclib and endocrine therapy in the treatment of HR‐positive, HER2‐negative mBC in the real‐world setting is similar to the efficacy reported in the PALOMA‐2 trial. Patients with lower neutrophil count may have a lower risk of early disease progression.

## Graphical abstract

The real‐world benefit of combination CDK4/6 inhibitor and endocrine therapy is similar to that reported in clinical trials. Treatment‐related neutropenia, assessed by absolute neutrophil count (ANC), may be correlated with lower risk of disease progression and a lower ANC threshold for dose reduction may be warranted.

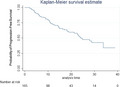

**Lay summary**

The real‐world benefit of combination CDK4/6 inhibitor and endocrine therapy is similar to that reported in clinical trials. Neutropenia, a common adverse event associated with CDK4/6 inhibitor therapy, may be associated with a lower risk of disease progression. We demonstrate that lower absolute neutrophil count (ANC) was significantly correlated with decreased risk of disease progression. A lower ANC threshold for CDK4/6 inhibitor dose delay and/or reduction may be warranted.

## 1 INTRODUCTION

Breast cancer is the most common cancer diagnosed in American women and is the second leading cause of death only after lung cancer.^1^ Most breast cancers are characterized by estrogen and/or progesterone receptor expression (hormone receptor [HR]‐positive) without overexpression of HER2 (HR+/HER2−), such that endocrine therapy is the backbone of treatment for metastatic disease.^2^, ^3^, ^4^ Current National Comprehensive Cancer Network (NCCN) guidelines recommend adjuvant endocrine therapy for all HR+ breast cancers, regardless of age, nodal status or treatment with systemic chemotherapy due to the tolerable toxicity profile, reduction in recurrence, and improvement in overall survival.^3^, ^5^, ^6^ Among postmenopausal patients with recurrent or de novo stage IV HR+/HER2− disease, combination therapy with a cyclin‐dependent kinase (CDK) 4/6 inhibitor and endocrine therapy (aromatase inhibitor or fulvestrant) is now standard of care. The PALOMA,^7^, ^8^, ^9^ MONARCH,^10^, ^11^ and MONALEESA^12^, ^13^ trials demonstrated significant improvements in progression‐free survival (PFS); several of these studies also show benefit in overall survival (OS) though additional studies are ongoing.^3^, ^7^, ^14^

Selective CDK 4/6 inhibitors, such as palbociclib, abemaciclib, and ribociclib are effective for breast cancers characterized by dysregulation of the cell cycle due to cyclin D1, which is activated by association with CDK 4/6 to permit cell cycle progression.^15^ Palbociclib is a first‐generation selective CDK 4/6 inhibitor and subject of the phase III PALOMA‐2 study of patients with advanced breast cancer.^7^ Patients treated with combination palbociclib and letrozole had improved PFS of 24.8 versus 14.5 months with combination placebo and letrozole (*p* < 0.001).

The most common adverse event (AE) with palbociclib therapy is dose‐dependent and dose‐limiting neutropenia.^16^, ^17^ The PALOMA‐1 and PALOMA‐2 trials reported serious (grade ≥ 3) neutropenia in 55% (*n* = 45) and 66% (*n* = 295) of patients, respectively, resulting in dose delay and/or reductions in 40% and 36% of patients.^7^, ^18^ A recent study by Wilkie et al. reported a similar AE profile and outcomes as PALOMA‐2 and concluded that dose reductions do not affect the efficacy of palbociclib.^7^, ^19^

Increased understanding of the immune system's role in oncogenesis has led to identification of neutrophils as mediators of pro‐tumor effects through two main mechanisms: angiogenesis and immune suppression. Angiogenesis is mediated through release of proangiogenic matrix metalloproteinases^20^, ^21^ and immune suppression is caused by inhibition of anti‐tumor CD8+ T cells.^20^, ^22^, ^23^, ^24^ Therefore, the improved survival from iatrogenic neutropenia may be caused by abrogation of the pro‐tumor effect exerted by neutrophils. The neutrophil–lymphocyte ratio (NLR) can be used as a surrogate measure of systemic inflammation and has also been correlated with improved breast cancer prognosis.^25^, ^26^, ^27^, ^28^, ^29^ These studies identify elevated NLR as an independent predictor of mortality and is associated with worse outcomes.

The primary objective of this study is to assess the real‐world benefit of first‐line CDK 4/6 inhibition for HR+/HER2− breast cancer in comparison to the results reported in the PALOMA‐2 trial.^7^ The secondary objective was an exploratory analysis to assess whether neutropenia, assessed by ANC and NLR, may be beneficial in the course of palbociclib therapy. In concert with the neutropenia resulting from CDK 4/6 inhibition, we hypothesize that there is a correlation between neutropenia and clinical outcome such that ANC and NLR can be used as biomarkers for breast cancer progression.

## 2 MATERIALS AND METHODS

### 2.1 Study design and study population

Institutional review board approval was obtained prior to the start of the study with waiver of consent. We conducted an observational study to investigate the effectiveness of combination palbociclib and endocrine therapy for the first‐line treatment of female breast cancer patients with advanced or metastatic HR+/HER2− disease treated at a single academic tertiary referral cancer center between 1 January 2015 and 1 March 2018. Patients were identified from a prospectively maintained registry tracking all patients treated with palbociclib. Blood count lab values were collected and used to calculate ANC and NLR, defined as the ratio of ANC to ALC. These lab values were obtained at baseline, every 2 weeks through the 8th week, then every 4 weeks through the 24^th^ week for a total of nine data points over 24 weeks. The normal range of ANC was 1,500–8,000 mm^‐3^ and normal range of NLR was 0.78–3.53. Only patients age ≥18 years who received combination palbociclib and selective endocrine therapy (letrozole, exemestane, or anastrozole) or fulvestrant as standard of care therapy for stage IV breast cancer and had all blood test results available for review were included in this study. Patients that received treatment on clinical trial or as second‐line or later therapy were excluded.

Demographic, clinicopathologic, and outcome data were collected from review of the electronic medical records. The data that support the findings of this study are available from the corresponding author, H.T.K, upon reasonable request.^30^

### 2.2 Objectives

The primary outcome was PFS after treatment with combination palbociclib and endocrine therapy. PFS was defined as the time from first palbociclib intake to the date of progression or death from disease, whichever occurred first. The secondary outcomes were correlation of ANC and NLR to PFS. The data used in this study reflect real‐world patients treated at a tertiary referral center. Therefore, disease progression was determined by each treating physicians’ assessment of the patient's treatment response.

### 2.3 Statistical methods

Descriptive statistics were used to describe demographic and clinical characteristics of our patient cohort. Continuous variables were summarized using median (interquartile range; IQR) and categorical variables were reported as count and percentage. A Wilcoxon signed‐rank test and a chi‐squared test were used to compare differences in continuous and categorical variables, respectively. In addition to the characteristics of the full cohort, we compared the subsets of patients with bone‐only metastases to patients with metastatic disease not confined to the bone (all other metastases). PFS was analyzed using Kaplan–Meier curves and log‐rank tests were used to compare PFS of patients with bone metastases and all other distant metastases. Univariable and multivariable Cox models were used to assess the correlation of ANC and NLR with clinical outcomes. Given that ANC varied with disease and treatment status, ANC was used as a time‐dependent variable in the Cox regressions. Instead of determining the risk group of a patient for the entire study based on the baseline ANC value, we treated ANC as a time‐dependent variable based on the ANC level at the time of progression to evaluate the risk group. Accounting for the time‐varying nature of covariates in the Cox model has more robust results because more information is utilized. A *p* < 0.05 was considered statistically significant for all tests. All analyses were performed using Stata 14.1 (StataCorp. 2015. *Stata Statistical Software*: *Release 14*.) and R version 3.5.2 (R Foundation for Statistical Computing).

## 3 RESULTS

A total of 165 predominantly Caucasian patients were included in this study. The median age at the start of palbociclib was 64 years (IQR 53–70). All patients received palbociclib as first‐line therapy in combination with endocrine therapy. Letrozole was the most used endocrine drug (*n* = 141, 86%); 20 patients were treated with fulvestrant (12%) (Table 1). Fifty‐four patients (33%) had bone‐only metastases and 111 (67%) patients had metastases at other sites not limited to bone. The median age of patients with bone‐only metastases (61 years, IQR 50–67) was slightly younger than that of patients with other metastases (65 years, IQR 56–70, *p* = 0.077). The median follow‐up time was 17.98 months (95% CI 14.89–20.93). The median ANC at baseline was 4.01 (IQR: 3.13–5.11) × 10^3^ mm^‐3^ and the median levels during the study period are presented in Table 2.

**TABLE 1:** Demographic and clinical characteristics of the study cohort

Demographic and clinical characteristics	*N* = 165	
	*N*	Percent or Median [IQR]
Age	165	64 [53, 70]
Endocrine therapy		
Anastrozole	3	1.8%
Exemestane	1	0.6%
Fulvestrant	20	12.1%
Letrozole	141	85.5%
Metastatic location		
Bone‐only	54	32.7%
All other metastases	111	67.3%
Absolute neutrophil count (×10^3^ mm^‐3^)		
Baseline	147	4.01 [3.13, 5.11]
Week 2	165	1.55 [1.14, 2.26]
Week 4	165	1.39 [0.94, 1.82]
Week 6	165	1.46 [1.11, 1.97]
Week 8	162	1.42 [1.11, 2.01]
Week 12	155	1.56 [1.16, 2.17]
Week 16	144	1.61 [1.11, 2.07]
Week 20	134	1.49 [1.16, 1.94]
Week 24	127	1.54 [1.22, 2.20]
Neutrophil–lymphocyte ratio		
Baseline	147	2.70 [1.86, 3.48]
Week 2	165	1.32 [0.89, 2.02]
Week 4	165	1.16 [0.81, 1.92]
Week 6	165	1.30 [0.91, 2.05]
Week 8	162	1.27 [0.87, 1.97]
Week 12	155	1.35 [0.98, 2.00]
Week 16	144	1.47 [0.92, 2.11]
Week 20	134	1.49 [1.00, 2.03]
Week 24	127	1.40 [0.99, 2.16]

Abbreviations: SD, standard deviation.

**TABLE 2:** Demographic and clinical characteristics of patients with bone‐only metastases and all other metastases

Variable	Bone‐only (*N* = 54)		All other metastases (*N* = 111)		*p* value
	*N*	Percent or Median [IQR]	*N*	Percent or Median [IQR]	
Age	54	12.10	111	11.00	0.145
Endocrine therapy					0.318
Anastrozole	0	0.00%	3	2.70%	
Exemestane	1	1.85%	0	0.00%	
Fulvestrant	5	9.26%	15	13.51%	
Letrozole	48	88.89%	93	83.78%	
Absolute neutrophil count (×10^3^/mm^3^)					
Baseline	48	3.70 [2.98, 5.11]	99	4.15 [3.27, 5.06]	0.268
Week 2	54	1.52 [1.00, 2.01]	111	1.56 [1.17, 2.39]	0.196
Week 4	54	1.30 [0.98, 1.66]	111	1.46 [0.92, 1.86]	0.526
Week 6	54	1.39 [1.08, 2.00]	111	1.48 [1.12, 1.92]	0.564
Week 8	54	1.42 [1.11, 2.16]	108	1.43 [1.11, 1.96]	0.904
Week 12	52	1.46 [0.97, 1.96]	103	1.57 [1.21, 2.20]	0.255
Week 16	47	1.36 [1.07, 2.13]	97	1.65 [1.17, 2.07]	0.263
Week 20	40	1.42 [1.16, 1.88]	94	1.53 [1.20, 2.01]	0.307
Week 24	37	1.54 [1.04, 2.20]	90	1.55 [1.24, 2.17]	0.592
Neutrophil–lymphocyte ratio					
Baseline	48	2.73 [1.82, 3.98]	99	2.68 [1.92, 3.44]	0.702
Week 2	54	1.27 [0.83, 1.90]	111	1.32 [0.90. 2.05]	0.513
Week 4	54	1.18 [0.75, 1.96]	111	1.16 [0.81, 1.92]	0.976
Week 6	54	1.36 [0.85, 2.11]	111	1.30 [0.94, 1.85]	0.912
Week 8	54	1.38 [0.83, 1.99]	108	1.17 [0.89, 1.94]	0.551
Week 12	52	1.30 [0.96, 2.03]	103	1.38 [0.98, 2.00]	0.892
Week 16	47	1.37 [0.86, 2.00]	97	1.48 [0.95, 2.26]	0.533
Week 20	40	1.45 [1.04, 1.96]	94	1.50 [1.00, 2.05]	0.930
Week 24	37	1.40 [0.96, 2.24]	90	1.43 [1.07, 2.14]	0.863

Abbreviation: SD, standard deviation.

**FIGURE 1:**
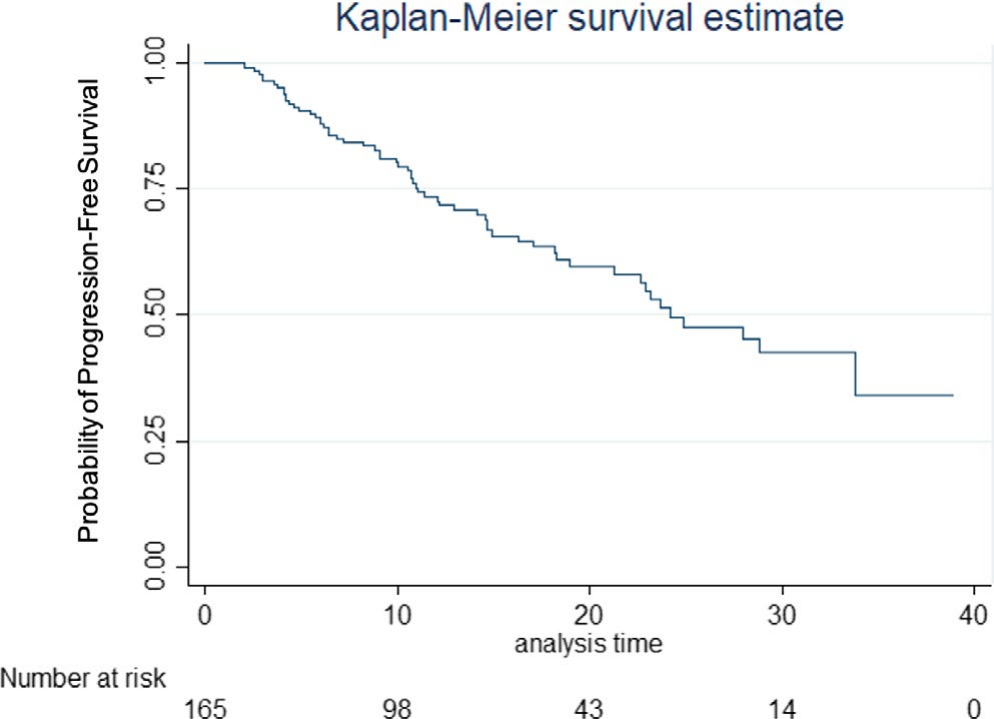
Progression‐free survival of patients treated with first‐line combination palbociclib and anti‐estrogen therapy

**FIGURE 2:**
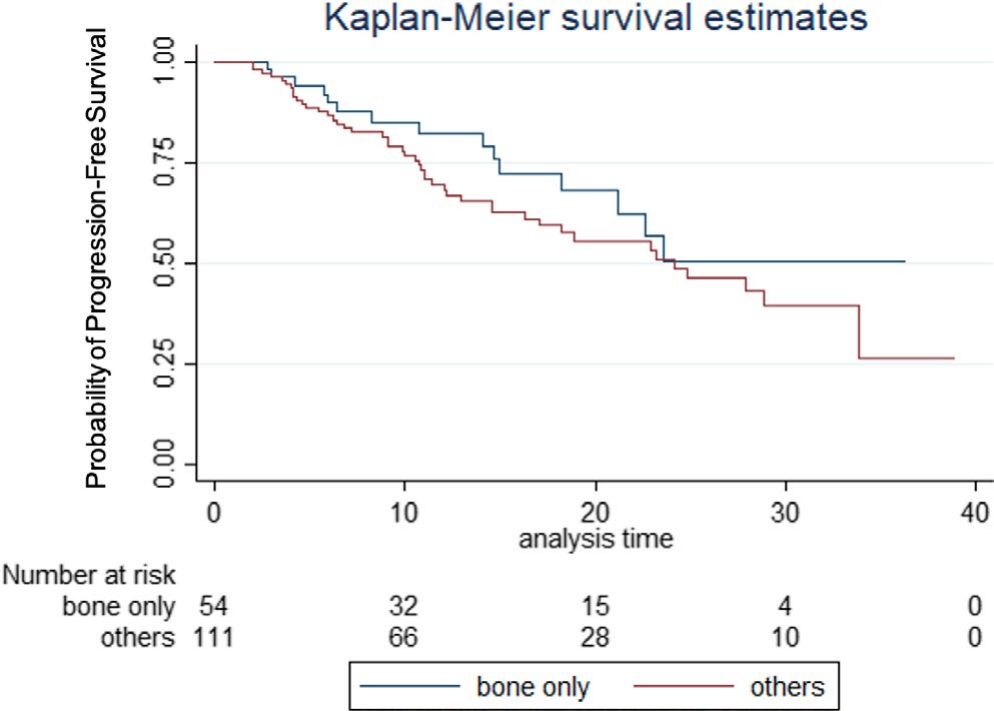
Progression‐free survival of patients with bone‐only metastases compared to all other metastases

The median PFS of the full cohort was 24.19 months (95% CI 18.93–NR) (Figure 1). The median PFS for patients with bone‐only metastases was not reached (95% CI 18.21–NR). The median PFS for patients with all other metastases was 24.19 months (95% CI 16.33–33.82). The log‐rank test showed no significant difference in PFS between patients with bone‐only metastases and patients with other metastases (*p* = 0.215) (Figure 2).

Univariable analysis showed that ANC was significantly associated with PFS (HR = 0.85; 95% CI 0.76–0.96, *p* = 0.009), meaning that a decrease in ANC is significantly associated with a lower risk of disease progression. This remained significant on multivariable analysis after adjustment for age, metastatic location, and type of endocrine therapy (HR 0.84; 95% CI 0.71–0.97, *p* = 0.008) (Table 3). NLR was also significantly associated with PFS on univariable analysis (HR = 1.11, 95% CI 1.01–1.21, *p* = 0.027), meaning that an increase in NLR is significantly associated with a higher risk of disease progression. However, this was not significant on multivariable analysis (HR = 1.07, 95% CI 0.97–1.18, *p* = 0.203). Baseline NLR and type of metastasis were not significantly associated with the risk of PFS.

**TABLE 3:** Cox regression models to assess the effect of absolute neutrophil count and neutrophil–lymphocyte ratio

Variable	Hazard ratio (95% CI)	*p* value	Hazard ratio (95% CI)	*p* value
	Univariable regression		Multivariable regression	
ANC (time‐dependent)^a^	0.85 (0.76–0.96)	**0.009**	0.84 (0.71–0.97)	**0.008**
NLR (time‐dependent)^a^	1.11 (1.01–1.21)	**0.027**	1.07 (0.97–1.18)	0.203
Baseline NLR	1.05 (0.93–1.19)	0.448	0.91 (0.79–1.06)	0.210
Metastatic location	1.45 (0.81–2.60)	0.217	0.73 (0.34–1.57)	0.418

Abbreviations: ANC, absolute neutrophil count; CI, confidence interval; NLR, neutrophil–lymphocyte ratio.

^a^ Adjusted for age, metastatic location, and type of endocrine therapy.

Bold indicates statistically significant values.

## 4 DISCUSSION

This study demonstrates that in real‐world clinical practice, on‐label use of palbociclib in combination with an aromatase inhibitor or fulvestrant demonstrates comparable efficacy with clinical trial results for the treatment of HR+/HER2− advanced or metastatic breast cancer. Our cohort is not the same as we allowed for all on‐label anti‐estrogen combinations while the PALOMA‐2 trial only included patients treated with letrozole, which was the most used aromatase inhibitor among our patients. Our cohort is more representative of the real‐world clinical setting where many different agents are utilized. Despite these differences, our cohort had a PFS of 24.2 months, nearly the same as the reported PFS in the PALOMA‐2 trial, which was 24.8 months.^7^

As previously mentioned, neutropenia is the most common AE with palbociclib therapy, with rates of dose reduction ranging from 16% to 20% as observed in other real‐world studies.^31^, ^32^ Due to previously described pro‐angiogenic and immunosuppressive effects of neutrophils, we hypothesized that the neutropenia associated with palbociclib therapy would lead to improved PFS. Through serial monitoring of ANC, we demonstrate that decreased ANC was significantly correlated with lower risk of disease progression, which remained significant even after adjusting for age, metastases, and type of endocrine therapy. For every 1 × 10^3^ mm^‐3^ decrease in ANC, we estimate a 15% decrease in the risk of progression. NLR also correlated with PFS on univariable analysis, but this correlation was not seen after multivariable analysis with adjustment for the same variables.

The PALOMA studies demonstrated significantly improved PFS among patients with metastatic disease. However, when comparing patients with bone‐only metastases to visceral metastases, patients with bone‐only metastases had a higher magnitude of benefit.^7^ Conversely, the MONARCH studies demonstrated significant survival benefit among patients with visceral metastases compared to bone‐only metastases, suggesting that among patients with bone‐only metastases, palbociclib should be the preferred CDK4/6 agent.^10^, ^11^ Our study did not identify differences in PFS between the two groups treated with palbociclib, perhaps reflecting differences in the cohort sizes.

To the authors’ knowledge, this study is the first to demonstrate positive correlation between ANC and clinical benefit with the combination of palbociclib and endocrine therapy. There are several other real‐world studies of this combination therapy with some differences. Kish et al. examined real‐world prescribing pattern, treatment adverse events, and provider compliance to monitoring guidelines 1 year after palbociclib approval. They demonstrated good adherence to blood count monitoring and similar grade 3/4 neutropenia rates compared with those observed in PALOMA‐2 and PALOMA‐3 trials.^31^ Waller et al. demonstrated favorable 6‐month PFS and 12‐ to 18‐month OS combination palbociclib and endocrine therapy.^32^ Another study by Varella et al. demonstrated a shorter PFS of 15.1 months in real‐world clinics compared with 24.8 months in PALOMA‐2 trial.^33^ Finally, a more recent study by Porte et al. reported similar median PFS in unselected patients who received palbociclib and endocrine therapy compared with published clinical trial data.^34^

We posit that the survival benefit of neutropenia is likely due to the suppression of pro‐tumor effects by neutrophils. Angiogenesis is mediated through degradation of the extracellular matrix by matrix metalloproteinase 9 (MMP‐9), which then causes release of potent angiogenic factors such as vascular endothelial growth factor (VEGF) and fibroblast growth factor 2 (FGF2).^35^, ^36^ Angiogenesis subsequently leads to tumor growth and progression. Neutrophil depletion has been shown to suppress this angiogenic effect.^36^, ^37^ The mechanism of T‐cell inhibition has been previously described in detail by Pillay et al.,^24^ but briefly, neutrophils inactivate T cell‐stimulating cytokines and induce shedding of IL‐2 and IL‐6 receptors. Next, reactive oxygen species and arginase lead to downregulation of TCRζ, arresting T‐cell growth and proliferation. Lastly, PD‐L1 expression is upregulated leading to T‐cell apoptosis. In a murine breast cancer model, Coffelt et al. demonstrated that neutrophil depletion did not affect primary tumor growth or histopathology, but instead resulted in a significant reduction of regional nodal and pulmonary metastases.^38^ Therefore, neutrophils have a significant role in tumor growth and metastasis and measurements of the neutrophil compartment are useful biomarkers to gauge patient outcome.^37^

CDK4/6 inhibitors have also been shown to directly affect cancer cells as well as indirectly via modulation of stromal cells, including T cells.^39^ Whether or not this immunomodulatory activity contributes to the clinical benefits of these drugs is unknown at this time. Based on our data showing correlation between neutropenia and clinical benefit, we hypothesize that the observed correlation may be due in part to a permissive microenvironment on T‐cell activation secondary to neutropenia.

Our study has the inherent shortcomings of a retrospective study and somewhat heterogenous treatment group due to the use of several different anti‐estrogen therapies. However, we do not believe the slight heterogeneity of the endocrine backbone affected our results since 86% of patients received letrozole as endocrine therapy. Other limitations include the fact that we did not collect data on comorbidities, performance status, dose delay and reductions, which could affect the outcome.

This study demonstrates that the degree of neutropenia during palbociclib therapy may correlate with PFS, and as such, we recommend reassessing the current practice of frequent dose delays and/or reductions based on an ANC threshold of 1,000 mm^‐3^. Further studies would be required to determine a safe threshold.

## 5 CONCLUSIONS

This study provides support for previously published clinical studies regarding the real‐world efficacy of on‐label CDK 4/6 inhibitor therapy for HR+/HER2− metastatic breast cancer. Furthermore, we have demonstrated a correlation of neutropenia, an adverse event from CDK 4/6 inhibitor therapy, as a possible biomarker of clinical benefit from CDK4/6 inhibitor therapy.

## CONFLICT OF INTEREST

R.L.B.C has received consulting honorarium from Bristol Myers Squibb, Pfizer, and Daiichi Sankyo. He has also received a research grant from Bristol Myers Squibb. The remaining authors have no relevant conflicts of interest to disclose.

## AUTHOR CONTRIBUTIONS

Xiaojun Zhong, Junjie Ma, Weihong Sun and Hung T. Khong: Study design conceived. Xiaojun Zhong, Weihong Sun and Hung T. Khong: Patients identified. James Sun and Xiaojun Zhong: Data collection. Junjie Ma: Statistical analysis. All authors contributed to analysis and interpretation of the data. Manuscript drafted by James Sun, Xiaojun Zhong, Junjie Ma, Weihong Sun and Hung T. Khong. All authors contributed to critical revision and approval of the final version of the manuscript.
